# Variation on a theme: pigmentation variants and mutants of anemonefish

**DOI:** 10.1186/s13227-021-00178-x

**Published:** 2021-06-19

**Authors:** Marleen Klann, Manon Mercader, Lilian Carlu, Kina Hayashi, James Davis Reimer, Vincent Laudet

**Affiliations:** 1 Marine Eco-Evo-Devo Unit, Okinawa Institute of Science and Technology, 1919-1 Tancha, Onna-son, Okinawa, 904-0495 Japan; 2 Present Address: Marine Eco-Evo-Devo Unit, Okinawa Institute of Science and Technology, 1919-1 Tancha, Onna-son, Okinawa, 904-0495 Japan; 3 Molecular Invertebrate Systematics and Ecology Lab, Graduate School of the Engineering and Science, University of the Ryukyus, 1 Senbaru, Nishihara, Okinawa, 903-0213 Japan; 4 Tropical Biosphere Research Center, University of the Ryukyus, 1 Senbaru, Nishihara, Okinawa, 903-0213 Japan; 5 Marine Research Station, Institute of Cellular and Organismic Biology (ICOB), Academia Sinica, 23-10, Dah-Uen Rd, Jiau Shi, I-Lan 262, I-Lan, Taiwan

**Keywords:** Pigmentation, Anemonefish, Variation, Mutants

## Abstract

Pigmentation patterning systems are of great interest to understand how changes in developmental mechanisms can lead to a wide variety of patterns. These patterns are often conspicuous, but their origins remain elusive for many marine fish species. Dismantling a biological system allows a better understanding of the required components and the deciphering of how such complex systems are established and function. Valuable information can be obtained from detailed analyses and comparisons of pigmentation patterns of mutants and/or variants from normal patterns. Anemonefishes have been popular marine fish in aquaculture for many years, which has led to the isolation of several mutant lines, and in particular color alterations, that have become very popular in the pet trade. Additionally, scattered information about naturally occurring aberrant anemonefish is available on various websites and image platforms. In this review, the available information on anemonefish color pattern alterations has been gathered and compiled in order to characterize and compare different mutations. With the global picture of anemonefish mutants and variants emerging from this, such as presence or absence of certain phenotypes, information on the patterning system itself can be gained.

**Supplementary Information:**

The online version contains supplementary material available at 10.1186/s13227-021-00178-x.

## Introduction

Body coloration, or pigmentation, plays an essential part in every animal’s survival strategy. Pigmentation is not only important for predator avoidance or protection against UV radiation, but also for reproductive success and more generally social interactions [10, 21, 33]. In fish, skin pigmentation depends on the distribution of pigment cells, also called chromatophores, which are derivatives of the neural crest, and are typically classified based on the colored pigments or types of crystals they bear as well as their ultrastructure [15]. The major teleost chromatophores are (1) brown blackish melanophores that contain melanin; (2) yellow/orange/red xanthophores/erythrophores that are distinguished by color and contain carotenoids and/or pteridines; (3) silver/iridescent iridophores which contain guanine crystals, and (4) white leucophores which bear uric acid.

Typically, chromatophore precursor/progenitor cells are established during embryonic development and set aside to later on contribute larval/adult chromatophores. In zebrafish (*Danio rerio*), currently the major model species for pigment pattern development in teleost fish, two distinct populations of precursors are formed, one for xanthophores and one for melano- and iridophores [5]. In medaka (*Oryzias latipes*), the xanthophore precursor is still multipotent and can produce either xanthophores or closely related leucophores [40]. In zebrafish, chromatophores must be formed continuously throughout their life to maintain their characteristic striped pattern [25, 45].

Patterns in general can be formed by two main principles: (1) cell autonomously by (genetic) pre-positioning cues or (2) by cell–cell communication [46]. The mechanisms for stripe formation (horizontal main axis) in zebrafish are relatively well understood. All three chromatophore cell types (melanophores, xanthophores and iridophores, no leucophores are present in zebrafish) interact and communicate to establish the adult color pattern of dark stripes and bright interstripes. Some evidence suggests that this mode of patterning follows the Turing model, according to which the number of stripes increases with the growth of the fish [41]. Interactions between zebrafish chromatophores include long-range as well as short-range effects [14, 46] such as: (1) iridophores attract melanophores in high numbers and induce their aggregation into prospective stripe areas (positive long-range); (2) iridophores exclude melanophores from interstripes (negative short-range), (3) xanthophores exclude melanophores from interstripes (negative short-range), and (4) positive and negative interactions between iridophores and xanthophores being required to confine the shape of interstripes (short range). Similarly, the addition of bars (vertical main axis) in the cichlid *Copadichromis azureus* requires cell–cell communication, with xanthophores and iridophores following the pattern of melanophore distribution [18].

Extensive zebrafish and medaka mutant collections have facilitated research on many aspects of pigmentation, such as fate specification, survival, proliferation, and differentiation [23, 24, 46]. In both taxa, researchers have mainly focused on embryonic phenotypes, and there is an extensive overlap of identified parallel phenotypes. The strategy of using mutants identified during large genetic screenings and then subsequently identifying the genes underlying their unusual phenotypes has therefore been very fruitful. However, such a strategy has been limited to established model organisms that are well set up in terms of mutagenesis, genetic screening, and experimental genetics [26, 47]. Although research on both zebrafish and medaka have effectively led to great scientific breakthroughs in our understanding of pigment pattern formation, they represent only a small fraction of living biodiversity, and therefore new Evo-Devo model species have been developed in the past few years. Among these newer models, anemonefishes are colorful coral reef fish that offer great promise to enlarge and deepen our knowledge of color pattern formation [54–56]. Anemonefish are relatively new marine model organisms and therefore scientific mutant screenings have not yet been performed, but pigmentation mutants are available thanks to aquaculture companies that aim to sell novel “fancy” fish for the aquarium market. Some of these anemonefish mutants are derived from rare variants that were observed and captured in the wild. Other mutants are the result of aquaculture crosses, either made on purpose or by accident. In this review, we aim to describe the main anemonefish mutants available in addition to natural variants, as they could be significant in providing clues on the underlying genetic mechanisms of pigment pattern formation in these fish. Here, the term “mutant” therefore refers to an established line from an aquaculture company, with the strain name written in parentheses. For these lines it is known that the observed pigmentation phenotype is based on a fixed genetic trait, or in other words, a mutation. Breeding schemes and outcomes detailed and provided on aquaculture companies’ websites can be used to infer heritability, although this will need to be genetically confirmed in the future. The term “variant” is used for wild individuals that display a coloration alteration, as it generally remains unknown if the phenotype is based on a fixed, inheritable mutation. However, often these wild variants are based on a mutation, since wild-caught aberrant individuals have served on several occasions as founders in the establishment of new strains in aquaculture (as mentioned above). Although there are some polymorphic traits in several species of anemonefish (numbers of bars, degree of melanism, bar regression with age), these will not be the focus of the present paper as these are complex situations that are often related to environmentally driven phenotypic plasticity. Studies of several of these polymorphic traits are currently underway in our laboratory.

## Color pattern formation in anemonefishes

Anemonefishes (subfamily Amphiprioninae) are a monophyletic group within the Pomacentridae (damselfish) family, comprising 30 species in two genera: *Amphiprion* (29 species) and *Premnas* (monospecific) [54]. They are well known for their ability to establish mutualistic symbiotic relationships with sea anemones and their brilliant color patterns have been proposed to be either a social display allowing species recognition [58] or an aposematic trait warning of harmful stings from their host [36].

**Fig. 1:**
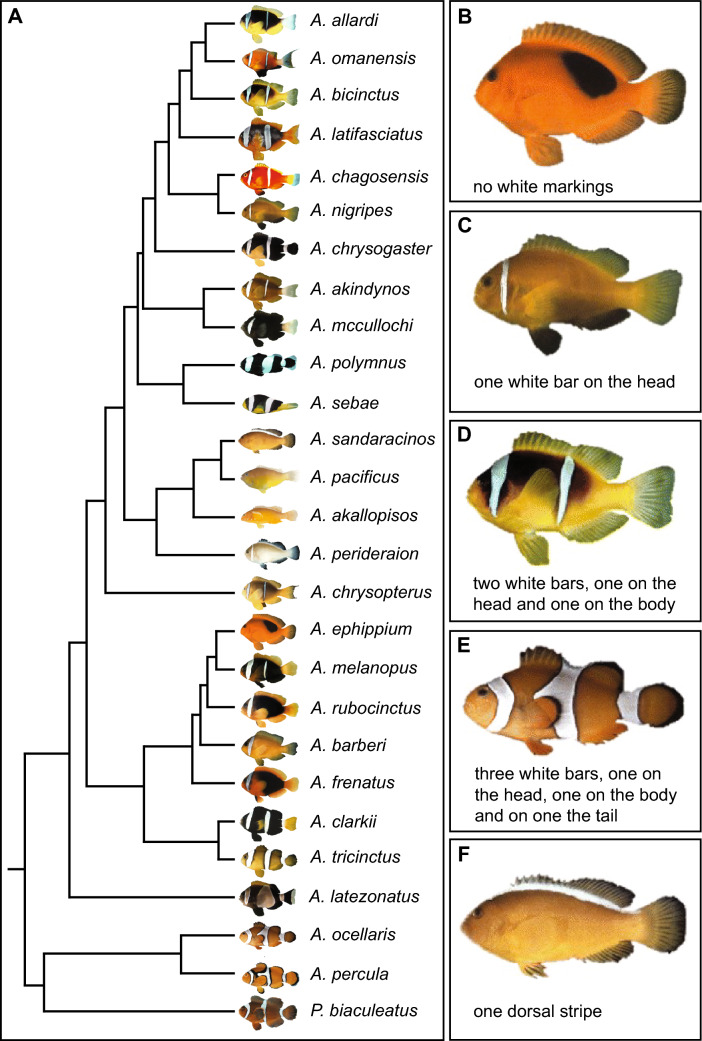
Phylogenetic relationships and categories of white bar appearance in adult anemonefishes. **A** Phylogenetic tree of 27 anemonefish species (adapted from the published work of Litsios et al. [31] and [52]. Three species could not be included into the tree because they are either rare with little genetic information available (*A. fuscocaudatus*) or hybrid species (*A. leucokranos* and *A. thiellei*). **B** No white bars; *A. ephippium*. **C** One white bar on the head; *A. nigripes*. **D** Two white bars, one on head and one on the body; *A. bicinctus*. **E** Three white bars, one on the head, one on the body and one on the tail; *A. ocellaris*. **F** Dorsal white stripe; *A. sandaracinos*

**Fig. 2:**
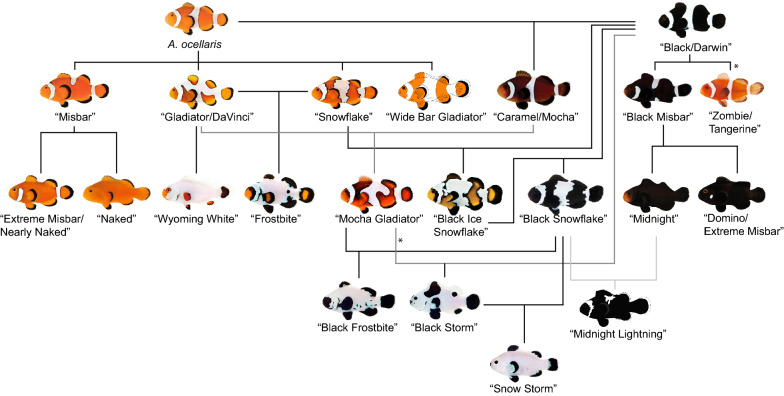
Common *A. ocellaris* strains and their origins. The information that has been used to create Fig. 2 is publicly available on the homepages of larger aquaculture companies in the US, such as ORA, S&R, and Proaquatix. The asterisks indicate a (spontaneous) mutation of the variants: a mutated “Mocha Gladiator” was used to establish the “Black Storm” retail line, while a mutated “Black” was used to create the “Zombie” retail line

**Fig. 3:**
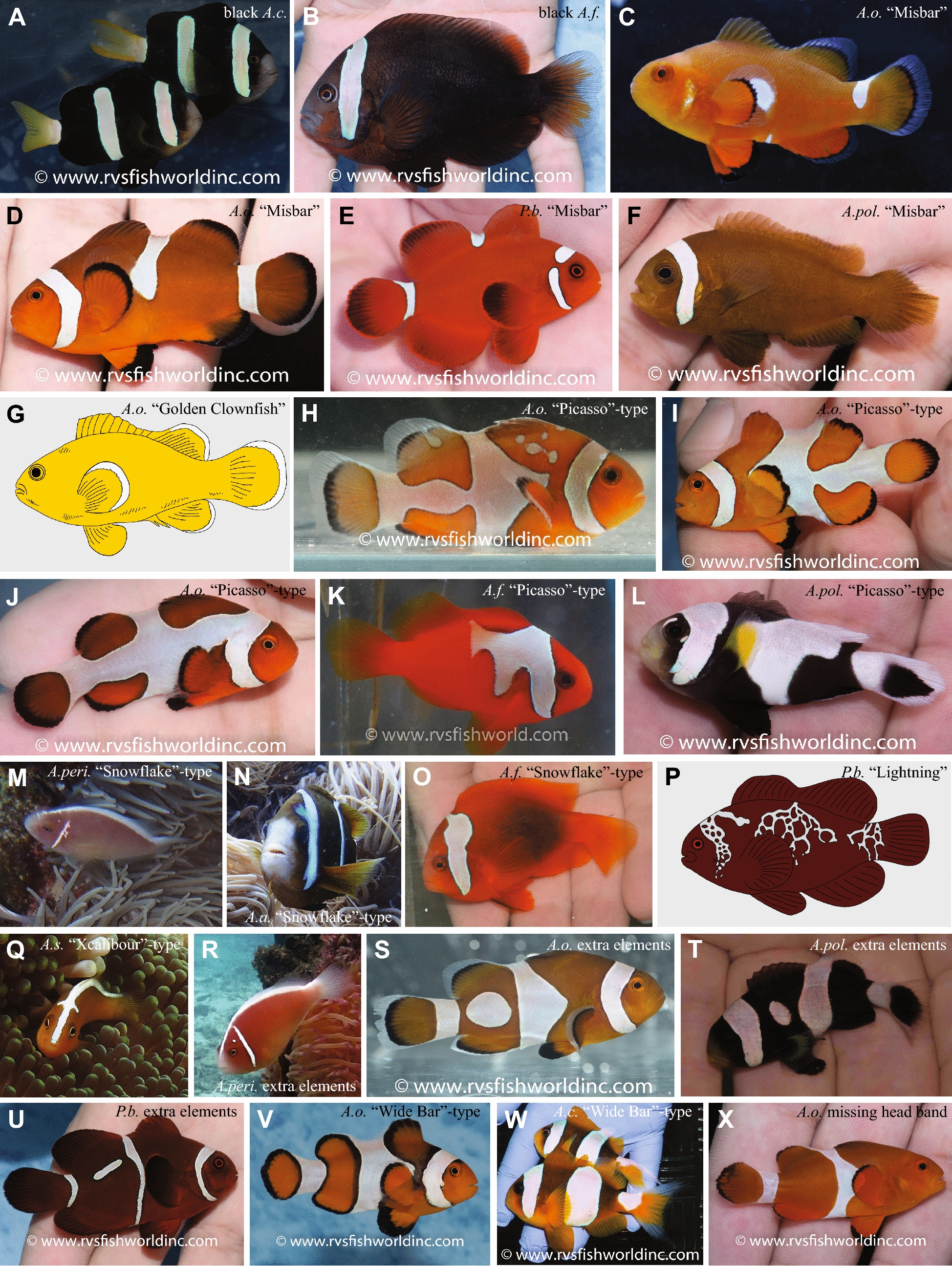
Color variants observed or taken from the wild. **A** Black *A. clarkii*, **B** Black *A. frenatus*, **C** Misbar *A. ocellaris*, **D** Misbar *A. ocellaris*, **E** Misbar *P. biaculeatus*, **F** Misbar *A. polymnus*, **G** “Golden Clownfish”, **H** “Picasso”-typed *A. ocellaris*, **I** “Picasso”-typed *A. ocellaris*, **J** “Picasso”-typed *A. ocellaris*, **K** “Picasso”-typed *A. frenatus*, **L** “Picasso”-typed *A. polymnus*, **M** “Snowflake”-typed *A. perideraion*, **N** “Snowflake”-typed *A. akindynos*, **O** “Snowflake”-typed *A. frenatus*, **P** “Lightning” *P. biaculeatus*, **Q** “Xcalibour”-typed *A. sandaracinos*, **R** *A. perideraion* with extra elements, **S** *A. ocellaris* with extra elements, **T** *A. polymnus* with extra elements, **U** *P. biaculeatus* with extra elements, **V** “Wide Bar”-typed *A. ocellaris*, **W** “Wide Bar”-typed *A. clarkii*, **X** *A. ocellaris* with missing head bar. Photo credit: A–F, H–L, O, and S–X were kindly provided by RVS Fishworld; G and P are schematic drawing made by MK; M and R were taken by KH; N was taken by MM, and Q was kindly provided by Ishigakijima Kamome Diving

**Fig. 4:**
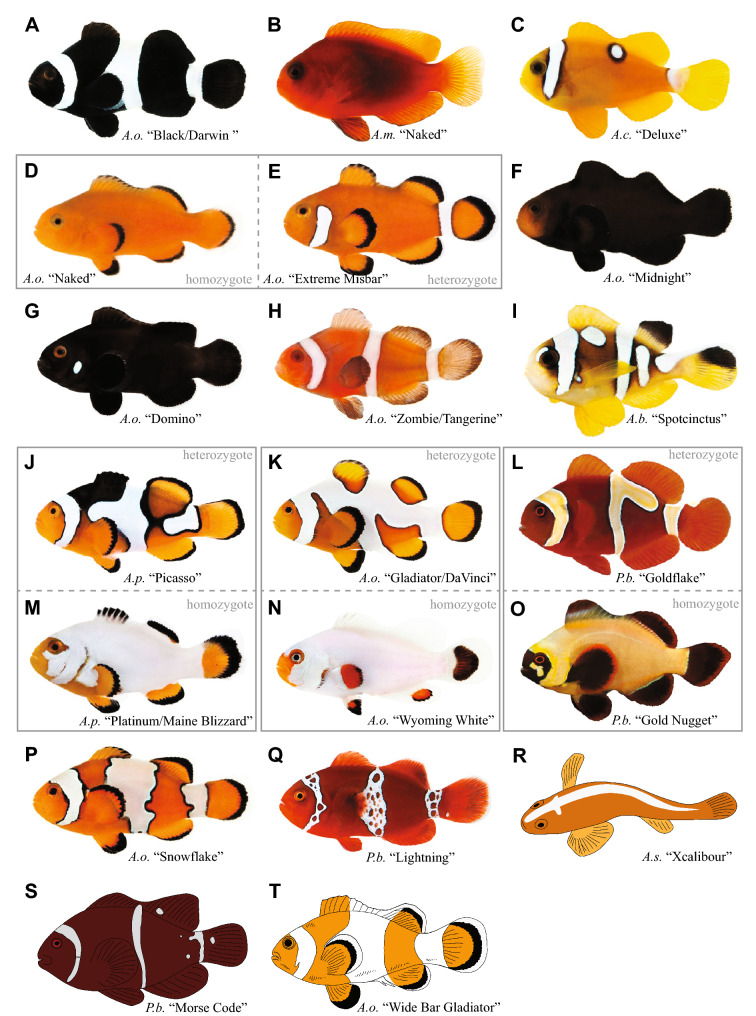
Color mutants available from aquaculture companies. **A** “Darwin Black” *A. ocellaris,* **B** “Naked Cinnamon” *A. melanopus,* **C** “Deluxe Clarkii” *A. clarkii,* **D** “Naked” *A. ocellaris,* **E** “Extreme Misbar” *A. ocellaris,* **F** “Midnight” *A. ocellaris,* **G** “Domino” *A. ocellaris,* **H** “Zombie” *A. ocellaris,* **I** “Spotcinctus” *A. bicinctus,* **J** “Picasso” *A. percula,* **K** “Gladiator” *A. ocellaris,* **L** “Goldflake” *P. biaculeatus,* **M** “Platinum” *A. percula,* **N** “Wyoming White” *A. ocellaris,* **O** “Gold Nugget” *P. biaculeatus,* **P** “Snowflake” *A. ocellaris,* **Q** “Lightning” *P. biaculeatus,* **R** “Xcalibour” *A. sandaracinos,* **S** “Morse Code” *P. biaculeatus,* **T** “Wide Bar Gladiator” *A. ocellaris*. Apart from the schematic drawings in **R**–**T** all images were provided by ORA

Adult anemonefishes display a relatively simple and robust color pattern comprising zero to three vertical white bars usually with a black outline on an orange-to-red body (Fig. 1A). Interspecific variations manifest mainly in the number of white bars, as well as the color of the body and the fins [31, 52, 58]. This characteristic color pattern has been classified into four different categories according to the appearances of adults: (1) species without bars (Fig. 1B); (2) species with one bar on the head (Fig. 1C); (3) species with two bars, one on the head and one on the body (Fig. 1D), and (4) species with three bars, one on the head, one on the body and one on the caudal peduncle/tail (Fig. 1E) [58]. Moreover, a few species of anemonefish show pronounced polymorphism (regarding the numbers of bars as well as background color), and four species exhibit a long dorsal stripe (Fig. 1F) [58]. This diversification can be traced back to successive evolutionary losses of bars from posterior to anterior. Bar formation in various anemonefish species is clearly developmentally constrained, and no species exhibit a single bar on the body or tail only, and there is always either a head bar only, or head and body bars, respectively. Moreover, there is an anterior to posterior sequence of bar acquisition during larval/juvenile development, but some anemonefish species subsequently lose the posterior-most bar(s) during adult color maturation [58]. We have recently shown that the adult pigmentation pattern of *A. ocellaris* and *A. percula* are, as in zebrafish [35], under the control of thyroid hormones, but with the distinct difference that in both anemonefishes the main target of these hormones seems to be the iridophores responsible for white bar formation [57]. All this suggests that there is an underlying robust patterning system that couples bar formation with antero-posterior cues, but the nature of this patterning system is currently unknown.

As for other fish, the larval pigmentation pattern of anemonefishes (comprising one or two horizontal stripes of single stellate melanophores) is very different from the adult pattern. Because the adult bar pattern does not change or adjust with size but follows an anterior–posterior sequence [58], it is highly unlikely that it develops in a Turing-like fashion. For *A. ocellaris* it has been shown that three chromatophore subtypes contribute to the mature body coloration: (1) orange skin color results from xanthophores with interspersed round melanophores; (2) white skin comprises iridophores and interspersed stellar melanophores, and (3) black skin is made of a dense number of round melanophores [55, 56, 58]. Iridophores are not only responsible for providing the white color but seem to be necessary for the correct positioning of melanophores as well. Treatment of *A. ocellaris* juveniles with TAE 684 (an inhibitor of tyrosine kinase receptors *alk* and *ltk*, known to be expressed in iridophores) results in a dose dependent loss of the bars—white color and black outline. This indicates that melanophores depend on iridophore signaling for their correct assembly and migration to form the adjacent black border. This hypothesis is supported by the observation that during white bar formation iridophores appear at the location of the prospective bar and push back the melanophores present in this area so that they create a black edge [58]. However, since TAE 684-treated larvae possess melanophores (intermingled with xanthophores in the orange-colored areas and forming a normal pattern on the fins) general development and survival is evidently not negatively affected by the lack of iridophores.

Even though iridophores often provide silver/bluish-greenish reflective color, in anemonefishes they mainly contribute white. This is due to the different arrangement of the guanine crystals present. When crystals are aggregated in platelets that are precisely organized, they give off an iridescent color, while they appear whitish when the crystal platelets are less organized as incoming light is scattered in various directions [15, 59]. In *A. ocellaris* the majority of iridophores contained platelets that are arranged parallel to one another in discrete stacks organized concentrically around the nucleus [55, 56]. In zebrafish it has been recently shown that there are different subtypes of iridophores that are part of stripes and interstripes [17]. Our own recent observations in anemonefish also suggest that distinct types of iridophores may exist, although their respective distributions and functions in the final pattern are not yet clear (M. Miyake, M.K and V.L, unpublished observation).

## Aquaculture of anemonefishes

Anemonefishes comprise a large proportion of the ornamental fish trade, and can be wild-caught or captive-bred. Many species reproduce regularly in captivity and survival rates are relatively high when rearing and feeding are optimized [44, 53]. *A. ocellaris* is among the five most-imported ornamental fish species in the US [49]. As with most domesticated animals, selective breeding efforts have been used to establish different strains for ornamental purposes. Several aquaculture companies have focused on anemonefish breeding and have produced various color mutations/alterations (see below). Due to the higher survival rate of anemonefish larvae in aquaculture compared to in the wild, there is a higher chance of mutant fish being successfully raised, if the mutation is not lethal.

Many variations are referred to using the same name by most companies, which makes it easier for the customer (and researcher) to navigate. However, some mutations have different names, such as “Gladiator/DaVinci” or “Platinum/Maine Blizzard”, and thus care must be taken. While a few mutations are restricted to specific companies and are often hard to find (such as “Xcalibour” or “Galaxy”), many are widely available within the trade. Continuing ongoing breeding efforts by several companies will surely lead to many more retail lines in the future. To date more than 40 color pattern variations/mutations are available, most prominently of *A. ocellaris*, *P. biaculeatus*, *A. percula* and *A. clarkii* (Additional file 1: Table S1). The scheme in Fig. 2 displays common *A. ocellaris* strains and their origins. The information that has been used to create Fig. 2 is publicly available on the homepages of larger aquaculture companies in the United States, such as ORA (https://www.orafarm.com/products/fish/clownfish/), S&R (https://www.seaandreef.com/marine-ornamental-fish/clownfish/), and Proaquatix (https://www.proaquatix.com/clownfish/). Some strains, like “Snow Storm”, are the result of crossings running for several generations and are usually associated with a higher retail price and often lower numbers of available individuals. It is noteworthy that many color mutations can be combined with morphological mutations. This is for example the case of “Longfin” that has been introduced in many different color mutants, such as “Domino” (“Longfin Domino”), “Black Storm” (“Longfin Black Storm”), or “Black Ice” (“Longfin Black Ice”).

In aquaculture, food plays an important role in fish coloration [64]. The skin color of captive-bred *A. ocellaris* juveniles can be enhanced when providing diets that contain natural sources of carotenoids [43]. It is for this reason that many aquaculture companies add carotenoids as supplements to fish food. Astaxanthin, β-carotene, and canthaxanthine are often used as supplements, but in *A. ocellaris* it has been shown that each carotenoid type present in the skin varies with regard with the dietary supplements [65]. Apart from coloration, it has also been shown that diet and fish density (and other ecological parameters) can affect white bar formation in *A. percula* and *A. ocellaris*, leading to the so-called “Misbar” phenotypes [2, 9]. Therefore, it is not always easy to determine if a given color variant has a genetic or environmental basis, and this is particularly relevant for fish taken from the wild.

## A sort of material and methods: characterization of color mutants/abnormalities in anemonefishes

This section describes and categorizes known mutants or abnormalities in coloration of different anemonefish species found in the wild or in the aquarium trade. The main source for wild anemonefishes with abnormal coloration have been companies that export wild specimens, such as RVS Fishworld or others, which are often featured on the internet site reefbuilders.com. Additionally, the photograph-sharing platform “flickr” (https://www.flickr.com/explore) was searched for images that were tagged as “clownfish”. Similarly, the “iNaturalist” (https://www.inaturalist.org/) website was searched for “*Amphiprion*” and “*Premnas*”, respectively. Detailed information (species, location, and category of alteration) and a link is given for all wild individuals that were recovered (Additional file 2: Table S2) as well as for many retail lines (Additional file 1: Table S1). Due to copyright restrictions, it is not possible to illustrate some of the natural variants, and in these cases schematic drawings are provided instead (e.g., Fig. 3G and P, Fig. 4R–T).

It must be noted that species identification from images can be erroneous and should therefore be regarded with caution, particularly for species that are difficult to distinguish in the wild (for example *A. frenatus* and *A. melanopus*) or for species that are closely related and can be hybridized in captivity. For example, *A. ocellaris* and *A. percula* are allopatric sister species that can be hybridized in captivity (“Snow Onyx” or “Black Photon”) and it can be difficult to distinguish between them based only on pictures.

Since images of adult individuals were primarily retrieved from the websites, the classification of pigmentation pattern abnormalities presented here is based on the adult phenotype only. This is in contrast with classifications established in zebrafish or medaka, which are typically based on embryonic and larval pigmentation patterns [23, 24]. Within the text rare natural variants and commercial aquaculture lines are presented together because the phenotypes are often similar to each other. However, for clarity, two separate figures are presented, one detailing wild variants (Fig. 3), and the other commercial lines (Fig. 4).

From the analyses of all these scattered data, the first conclusion is readily apparent; that two broad categories of pigmentation phenotypes can be recognized in adult anemonefishes, namely (1) imbalance of chromatophore subtypes, and (2) irregular patterning mechanisms. We will therefore describe and discuss these two categories successively.

### Class I: imbalance of chromatophore subtypes

A very prominent example is provided by geographically restricted wild melanistic populations, where the orange color is replaced by black color, resulting in black anemonefishes with white bars as seen in (1) completely black *A. ocellaris* found around Darwin, North Australia, (2) completely black *A. clarkii* from Ogasawara Island (Japan) and the Philippines (Fig. 3A), (3) completely black *A. frenatus* from southern Japan and the Philippines (Fig. 3B), and (4) partially black *A. percula* found around the Solomon Islands. All retail lines for “Black/Darwin” *A. ocellaris* (Fig. 4A) are offspring from wild-caught individuals, while the “Onyx” *A. percula* retail lines come from selective breeding efforts. Even though all these individuals do not appear to lack a specific chromatophore subtype, the number of melanophores is highly increased, and dominates the color of nearly the entire fish, apart from the white bars.

Many wild individuals with reduced numbers of iridophores and often decreased black edging are known, leading to the so-called “Misbar” phenotype. The degree of mis-barring is highly variable, typically only affecting one of the bars, predominantly the body bar. As mentioned above, this phenotype can be linked to environmental factors, as well as to mutations. Several anemonefish species show this rather common phenotype and are widely distributed: *A. ocellaris* (Fig. 3C, D), *P. biaculeatus* (Fig. 3E) and *A. polymnus* (Fig. 3F) from the Philippines, *A. bicinctus* from the Red Sea, *A. chrysopterus* from the Marshall Islands, *A. melanopus* from the Coral Sea, and *A. clarkii* and *A. percula* from the Solomon Islands. Both wild *A. melanopus* and *A. clarkii* individuals have been employed to establish the respective retail lines “Naked Cinnamon” (Fig. 4B) and “Deluxe Clarkii” (Fig. 4C). For *A. ocellaris*, retail lines come from selective breeding of a few founder individuals with severely reduced numbers of iridophores. “Naked” *A. ocellaris* (Fig. 4D) display a solid orange body with no white bars and no black outline, and are believed to be homozygous for a mutation of an unknown gene. “Misbar” and “Extreme Misbar” *A. ocellaris* (Fig. 4E) retain some white color in the position of the head, body and/or tail bar and are supposed to represent the heterozygous state of the same mutation. These assumed genetic states need to be confirmed scientifically, and for now have been inferred from breeding outcomes provided by ORA. For example, if “Misbar”x”Misbar” are crossed most offspring will be “Misbar”, some will be “Naked” and occasionally there will be wild-type fish (ORA, personal communication). If the body color is black instead of orange, the retail lines are called “Midnight” (Fig. 4F) and “Domino” (Fig. 4G), respectively. The “Midnight” strain arose as a separate mutation and is not the result of a specific crossing scheme involving “Naked”.

There is only one known wild-caught individual, from Indonesia (most likely *A. ocellaris*), that lacks both melanophores and iridophores—known as the “Golden Clownfish” (Fig. 3G). The absence of all melanophores, including the black outline of the white bars, the fins and the orange-colored areas is striking. This contrasts with individuals that lack iridophores only, because these individuals exhibit normal black coloration of the fins and orange-colored areas (see “Naked” *A. ocellaris* Fig. 4D). It is unclear if a retail line has been completely established yet, but Similan Farms Clownfish has a strain labeled “Super Yellow Percula” (showing very limited black coloration in fins) that is reminiscent of this phenotype.

A special case is presented by the hypomelanic *A. ocellari*s that are called either “Zombie” (Fig. 4H) or “Tangerine” in the aquarium trade. To the best of our knowledge, this phenotype has not been discovered in the wild yet. Although melanophores are most likely present in this color morphotype, no or very little melanin (black color pigment) is produced.

### Class II: irregular patterning mechanisms

Variations in bar shape occur regularly, are highly variable between individuals, and vary greatly in their extent – from a small bulge to extensive shape deformations. In general, there seem to be several different genetic mutations underlying these patterning abnormalities.

The most commonly observed mutation results in bars that are interconnecting, with an irregular but smooth outline, and often also include extra white markings in between bars. Wild examples include *A. ocellaris* (Fig. 3H–J), *A. percula*, *A. frenatus* (Fig. 3K), and *A. polymnus* (Fig. 3L). The *A. percula* retail line is called “Picasso” (Fig. 4J), and more and more species have been bred showing “Picasso”-type mutations, such as *A. ocellaris* “Gladiator/DaVinci” (Fig. 4K), *A. clarkii* “Galaxy”, *A. bicinctus* “Spotcinctus” (Fig. 4I), *A. seba*e, and *P. biaculeatus* “Goldflake” (Fig. 4L). For *A. percula* “Picasso”, *A. ocellaris* “Gladiator/DaVinci”, and *P. biaculeatus* “Goldflake” it is known that heterozygous mutants exhibit the described phenotype, while homozygous mutants display a nearly complete expansion of the white color on the body and are called “Platinum/Maine Blizzard” (Fig. 4M), “Wyoming White” (Fig. 4N), and “Gold Nugget” (Fig. 4O), respectively. As discussed above, these assumed genetic states need to be confirmed scientifically, and have been inferred from breeding outcomes provided by ORA. For example, if “Picasso”x”Picasso” are crossed, offspring will comprise “Picasso”, “Platinum” and wild-type fish. Similarly, if “Gladiator”x”Gladiator” are crossed the offspring will include “Gladiator”, “Wyoming White” and wild-type fish. Even though viability is low, all offspring of “Gold Nugget”x”Gold Nugget” will be “Gold Nugget”.

A second mutation is characterized by irregular white bars with exaggerated and jagged edges. In the aquarium trade, this phenotype is known as “Snowflake” and is only known in *A. ocellaris* (Fig. 4P). There are few wild individuals known with similar phenotypes, but the altered area is comparatively small as seen in *A. perideraion* (Fig. 3M), *A. akindynos* (Fig. 3N), and *A. frenatus* (Fig. 3O).

A third mutation, only known from *P. biaculeatus*, results in a network-like disruption of all three white bars, that often also spreads over the body, and is called “Lightning”. This mutation has been found mainly in Papua New Guinea, and wild founder individuals (Fig. 3P) have been used for all retail lines available (Fig. 4Q).

A special case is presented by a phenotype found only in *A. sandaracinos*, which is characterized by a cross-like mark (a horizontal branching of the dorsal stripe) just posterior of the eyes. *A. sandaracinos* usually has no bars, and only a single dorsal stripe. However, in this morphotype it appears as if a head bar has started to form in the dorsal-most region only. Contrary to all other bar shape alterations, this phenotype is very reproducible between individuals in the wild (Fig. 3Q), as well in the aquarium trade where they are called “Xcalibour” (Fig. 4R).

Occasionally, bars are interrupted with dot-like patterns, or there are white dots displayed in areas where there usually is no bar, as found in *A. perideraion* (Fig. 3R), *A. frenatus, A. ocellaris* (Fig. 3S), *A. polymnus* (Fig. 3T), and *A. percula*. Several wild-caught *P. biaculeatus* individuals from Papua New Guinea exhibited extra dots and even dashes between the bars or emerging from bars (Fig. 3U), and have been used to establish the retail line “Morse Code” (Fig. 4S).

Another type of altered patterning is individuals showing the normal arrangement and shape of bars, but with an increased width. In the aquarium trade these are known as “Wide Bar Gladiator” in *A. ocellaris* (Fig. 4T). A few wild *A. ocellaris* (Fig. 3V) individuals are known, as well as a local population of *A. clarkii* in the Philippines (Fig. 3W). It should be mentioned that the normal color pattern of *A. latezonatus* displays an exceptionally wide middle bar.

## Discussion: anemonefish pigmentation mutants in a wider context

The scattered information regarding wild and commercial pigmentation mutants of anemonefishes has been gathered in this work, and compiled and categorized into two classes, encompassing either chromatophore imbalances (class I) or patterning irregularities (class II).

What global picture emerges from these data? Interestingly, although several classes of mutant phenotypes can be identified, there are several potential phenotypes that do not seem to exist, predominantly the complete absence of chromatophore subtypes (lack of xanthophores or lack of all chromatophores). In order to learn something about pigmentation pattern formation, biological processes that could lead to the observed mutant phenotypes must be taken into consideration and investigated in detail. Even though much more data, especially experimental data, are needed to perform these analyses, we will try, in the following discussion, to link selected anemonefish mutant phenotypes to potential processes affected.

### What can we learn about color pattern formation from pigmentation mutants?

Classification of pigmentation phenotypes in zebrafish and medaka has allowed researchers to clarify which biological process(es) might be affected by the underlying mutation. As pigmentation heavily relies on chromatophores and their spatial arrangement, the neural crest development is of particular interest. Key developmental processes are (1) specification, (2) proliferation, (3) differentiation, (4) survival, and (5) patterning [23]. Disruptions in any of these have the potential to alter pigmentation patterns.

#### Chromatophore specification

Defects during specification are likely present if chromatophore numbers are strongly reduced, but the remaining cells appear normal and in the correct position.

Mutations related to “Naked” phenotypes (highly reduced white barring, Fig. 4B–G) are most likely caused by genes responsible for iridophore development, in particular specification. As mentioned above, the reduction of iridophores will indirectly reduce the number of melanophores that form the edge of the white bars, but melanophore distribution remains normal in fin and body coloration. In zebrafish, several genes have been linked with iridophore specification and they can serve as an entry point to investigate “Naked”-typed mutants. For example, *Foxd3* expression is required for neural crest derivatives to develop into iridophores [29]. *Leucocyte tyrosine kinase* (*ltk*) controls iridophore establishment, proliferation, and survival, and is associated with at least two mutants, “shady” (lack of iridophores) and “moonstone” (ectopic iridophores) [13]. Endothelin receptor signaling is crucial for larval iridophores highlighted by the importance of genes encoding *Edn3b* (*Endothelin 3b*) and *Ednr3b* (*Endothelin receptor b1a*) [28].

Apart from iridophores, other chromatophore subtypes might be also affected, as demonstrated in zebrafish (e.g.: the “nacre” mutant does not possess melanophores) and guppies (e.g.: the “blue” mutant lacks xanthophores). However, in anemonefish specification defects of either melanophores or xanthophores have not been discovered yet, neither in the wild (apart for the “Golden Clownfish”, Fig. 3G), nor in aquaculture. Even though some retail mutant strains are nearly or completely white (*A. ocellaris* “Wyoming White”, Fig. 4N, A*. percula* “Platinum/Maine Blizzard”, Fig. 4M) or completely black (*A. ocellaris* “Midnight”, Fig. 4F) as adults, larvae and juveniles of these lines display an orange body coloration that is slowly replaced by a different color during maturation (own personal observation, correspondence with aquaculture companies). The face and in particular the region around the mouth is usually the last to change color as shown in the “Midnight” mutant (Fig. 4F). The “Golden Clownfish” (Fig. 3G) is the only known individual that appears to lack both iridophores and melanophores, leaving only xanthophores to color the entire body uniform yellow.

All three chromatophore subtypes are affected when genes required for normal development of neural crest derivatives are mutated, such as mutations of *Sox10* (*SRY-related HMG-box 10*). The corresponding zebrafish mutant is called “colorless” [23] and the same gene is also required for chromatophore development in guppies [12]. To the best of our knowledge, no anemonefish lacking all three chromatophore subtypes has yet been found. Neural crest derivatives do not only specify chromatophore subtypes, but also craniofacial cartilage, peripheral and enteric neurons as well as glia [4, 22]. It is possible that in anemonefish mutations that affect these additional cell types are lethal and therefore have not been identified so far.

Of course, it is difficult to go further with analyses of the existing mutants without more detailed information. But from the data available so far, very interesting and focused questions emerge: Do xanthophores and melanophores in anemonefish or rather their precursors have other functions that if disrupted lead to highly increased mortality? Alternatively, it may be that the specification process for these cells can take alternative routes, so even if one gene is knocked out, others can replace it so that the precursor is still established. But why should this situation be different for iridophores? All these questions are awaiting molecular and developmental characterization of these specification mutants.

#### Chromatophore proliferation

Proliferation defects can also be indicated by a strongly reduced number of chromatophores as well as normal and correct positions of the remaining chromatophores.

Many retail lines display a pronounced shift towards either partially or completely white or black morphotypes. However, this does not necessarily mean that the establishment of the xanthophore lineage is negatively affected. For example, larvae and juveniles of many of these lines (such as “Darwin Black”, “Black Storm”, “Snow Storm”, and “Wyoming White”) usually display an orange body coloration that is slowly replaced by black color during maturation. This suggests that initial xanthophore specification and proliferation processes are unaffected, but their subsequent fate remains to be analyzed. Possible scenarios include (1) melanophores (or iridophores) replacing xanthophores; (2) xanthophores changing chromatophore subtype identity, or (3) xanthophores remaining present but becoming covered by other chromatophores. For example, anemonefish skin chromatophores are distributed within the epidermis as well as the dermis [55, 56] and it is possible that changes in the top layers cover the coloration of the deeper layers. In this hypothesis, it is assumed that xanthophores remain present in a deeper epidermal layer, while melanophores (or iridophores) spread in a more upper layer and therefore overlay the orange color. Regardless of the exact mechanism, proliferative processes of melanophores (or iridophores, respectively) are enhanced above normal levels during juvenile color maturation.

#### Differentiation processes

Alterations of cell differentiation processes can include a variety of phenotypes, such as reduced or altered pigmentation or abnormal cell morphology.

A first example for a differentiation defect is the “Zombie” mutation (Fig. 4H), which is most likely caused by the lack of melanin (albinism). In albinism, melanophores are present in their normal arrangement and distribution but they are de-pigmented, either completely lacking melanin or displaying highly reduced melanin levels [39, 63]. Since melanophores are present in these mutants, regulatory genes, such as *Mitfa* (*microphthalmia transcription factor a*) or *kita* (*receptor tyrosine kinase a*), are unlikely to be affected in these mutants. Genetic mutations in a gene involved in melanogenesis can potentially result in reduced or absent melanin synthesis leading to reduced pigmentation levels. The biosynthetic pathway during which L-tyrosine is converted to melanin through several enzyme catalyzed steps involves many genes, such as *MC1R* (*melanocortin 1 receptor*), *tyr* (*tyrosinase*), *tyrp1* (*tyrosinase-related protein-1*), and *dct/tyrp2* (*dopachrome tautomerase*). The most well documented example is *MCR1*, which contributes to melanin-related polymorphisms in several animal species, including the guppy *Poecilia reticulata* [61], zebrafish [51], and various vertebrates [19]. Another potential candidate is *oca2* (*oculocutaneous albinism II*). When its function is disrupted, the first step of melanin synthesis, the conversion of L-tyrosine to L-DOPA, is impaired, and this situation is responsible for the evolution of albinism in the cavefish *Astyanax mexicanus* [27]. Any of the above-mentioned genes might be responsible for the “Zombie” mutants in anemonefish. Interestingly, the first “Zombie” mutant first arose from a pair of “Black/Darwin” *A. ocellaris* (Fig. 4A). Juvenile “Zombie” mutants display a bright yellow-orange body coloration with white bars devoid of the black edge and have red eyes. Later on, individuals turn a darker shade of orange-reddish to reddish-brown. This suggests that some melanin is produced and accumulates, resulting in the darker shades of mature individuals. Because the original “Zombie” arose from completely black parents, melanophores cover the entire body and the little melanin that is produced is evenly distributed.

Another example of altered pigment cell differentiation is the icy blue, iridescent hue (instead of the normal white coloration) that is usually more prominent near black coloration. This altered pigmentation feature is most often seen in black and white morphotypes of *A. ocellaris* mutant lines, such as “Black Frostbite” or “Black Snowflake” (see also https://reefbuilders.com/2018/03/05/blue-clownfish/). It is noteworthy that some species of anemonefish such as *A. chrysopterus*, *A. frenatus*, *A. melanopus*, and *P. biaculeatus*, show an increased bluish appearance at the black border as part of their normal pigmentation patterns. This phenotype is most likely linked to the arrangement and organization of guanine platelets and crystals within the iridophores. As mentioned above, an iridescent color can be observed when crystals are aggregated in platelets that are precisely organized, while they appear whitish when the crystal platelets are less organized as light is scattered in various directions [15, 59]. Guanine platelet formation requires *gmps* (*guanine monophosphate synthetase*) expression [42] and a mutation in this gene could potentially alter platelet formation. However, since the blue-iridescent hue of iridophores usually appears near the black border, it is also possible that the presence of melanophores is partially responsible for guanine crystals to be orientated differently. Interestingly, the existence of two different subtypes of iridophores has been shown recently in zebrafish [17] and the authors suggest that the presence of melanophores might affect the patterning of both iridophore subtypes.

#### Pigmentation patterning

Patterning mechanisms are likely affected if a subset of the pattern is absent or altered in any way (e.g.: position, extra elements). In general, these changes are very hard to predict and will most likely affect tissues other than the neural crest.

In anemonefishes, major patterning factors concern the anterior–posterior succession of white bars. Both the position as well as the sequence of white bar appearance appears highly constrained. All species of anemonefish that possess white bars have them at the same position: (1) the transition of head to body; (2) the middle of the body, and (3) the caudal peduncle/tail. This suggests that the same morphological landmarks are used to establish the exact position of the bars. For the white body bar, it has been suggested that the transition between anterior spines and posterior soft rays of the dorsal fin acts as a pre-positioning cue [58]. More specifically, an indentation (made by longer anterior spines and shorter posterior spines) is more pronounced in species that have two or three white bars and less obvious in species with zero or one bar [58]. In addition to the exact position of the white bar, their developmental appearance is also highly conserved and constrained, as mentioned above. No species exhibits a single bar on the body or tail only. So far, only one wild-caught fish has been found that exhibits a disruption in this normal anterior–posterior patterning (Fig. 3X). This specimen lacked the head bar but showed normal formation of the body and tail bars. As there usually is an anterior to posterior sequence of bar acquisition during larval/juvenile development, this specimen is very fascinating, but unfortunately, its fate is unknown. Therefore, it is unclear if the underlying mechanisms are inheritable, and the molecular and cellular mechanisms responsible for the anterior to posterior sequence of bar appearance/formation remain to be analyzed.

Pigmentation research on anemonefishes offers the unique opportunity to study boundary formation—with melanophores forming a distinct border between the other two chromatophore subtypes, which is much harder to investigate in zebrafish or medaka (zebrafish: differently colored stripes, medaka: uniform color). It can therefore be assumed that novel processes will be revealed from such future analyses. Like the underlying mechanisms for anterior–posterior patterning, the genetic cause(s) for mutations that result in abnormal bar shape are rather hard to predict. The failure of different subtypes of chromatophores to communicate and interact could be responsible for altered boundary formation. Indeed, preliminary data suggests that melanophores restrict iridophore expansion (our own observation). Therefore, if melanophore regeneration and migration abilities are impaired, this could potentially lead to altered bar shapes. As outlined above, differences can be observed between several types of bar shape mutations. “Picasso”-typed mutations (Fig. 4I–L) are the most widespread and have been found in at least eight different species of anemonefish (*A. percula*, *A. ocellaris*, *A. clarkii*, *A. bicinctus*, *A. sebae*, *A. frenatus/A. melanopus*, *A. polymnus*, and *P. biaculeatus*). Moreover, the striking similarities between heterozygous and homozygous phenotypic appearances (compare Fig. 4J/4 M with 4 K/4 N with 4L/4O) in three species (*A. percula*, *A. ocellaris*, *P. biaculeatus*) suggests that the underlying genetic mechanisms are very similar, if not the same, while phenotypic appearance remains highly variable. Comparative developmental analyses of different species would be helpful to investigate when color alterations first appear and if they are similar in different species. Furthermore, genetic analyses of all species showing “Picasso”-typed mutations would show if the same genes are indeed affected.

Preliminary research on “Snowflake” *A. ocellaris* (Fig. 4P) suggest that a local decrease of melanophores forming the black edge might enable the iridophores to expand into neighboring areas, leading to the jagged black outline of this mutation (own observation).

### Ecological and evolutionary implications of color pattern variations

The ecological functions of color patterns in anemonefishes are still unclear, probably because experimental studies are scarce. However, it has been hypothesized that color might play a role in various functions, such as (1) concealment and camouflage either from predators [36, 58] or from resident fish in the case of juveniles [6], (2) species recognition and co-existence [58], and (3) individual identification [8]. Alteration of color pattern via mutation might then affect the adaptive value of the color pattern trait, and hence the fitness of the individual. Indeed, some color pattern mutations observed in the aquarium trade are never or very rarely observed in the wild, for example almost completely white morphotypes or individuals with no bands in species that usually have bands. This suggests that mutations which result in drastic color pattern alterations might have a negative effect on the survival of individuals in the wild and are therefore negatively selected against. However, other mutations are more widespread (like melanism, and “Picasso”-typed bar shape alterations), and individuals survive long enough to reach reproductive positions. This indicates that these mutations have no or negligible negative effects on survival, as the ascension to breeding positions is a lengthy process as juveniles go up the social hierarchy [7]. In the aquarium trade, *A. ocellaris* is by far the anemonefish species that exhibits the most color mutations, followed by *P. biaculeatus* (Additional file1: Table S1). In the wild, most mutant individuals are *P. biaculeatus* or *A. perideraion*, and have been noted particularly from within the Coral Triangle area. Ecological field studies within the Ryukyu Archipelago revealed relatively high rates of abnormal coloration in *A. perideraion* (1.8%) and *A. sandaracinos* (2.56%), but negligible rates for the remaining four anemonefish species present in this region (*A. clarkii*, *A. frenatus*, *A. ocellaris*, and *A. polymnus*) (Kina Hayashi, unpublished observation). From a limited Internet search using the photo sharing platform flickr (https://www.flickr.com/explore), a total of 36,195 pictures that were tagged as “clownfish” were screened for images of wild mutant individuals. Only approximately half of those images displayed natural marine environments, from which 34 aberrant anemonefish images were retrieved (0.18%). This figure is an only gross estimate and most likely represents a minimum of wild mutant anemonefishes. On the other hand, it may be that wild anemonefish variants are more noticeable and recreational divers might take more photographs of unusual individuals, and clearly more data from field observations are needed to better establish the true rates of abnormal coloration of anemonefish. As well, wild-caught specimens with aberrant color patterns are definitively targeted by export companies to be sold to aquaculture companies or private collectors [37].

From these observations, it can be concluded that most variants are rare and therefore selected against, implying that variations do not provide any advantages. It is possible that even though mutant individuals may reach breeding positions, the underlying mutation is usually not transmitted to an increased number of aberrant juveniles, that in turn would join or establish colonies. One reason could be that juveniles with color alterations are less successful in joining an existing colony precisely because they exhibit a different color pattern. The investigation of the ecological function(s) of color patterns would greatly benefit from experimental studies using mutants, which could provide new insights on this poorly understood aspect of anemonefish ecology. For example, in mesocosm experiments it would be interesting to study how juveniles with different white bar patterns are differentially affected during the recruitment phase when joining an existing colony. Moreover, the effect of changes in bar patterns on the aggressiveness of conspecifics could also be studied. These types of analyses are currently underway in our laboratory.

Finally, it should be noted that some polymorphic variations in bar shape are observed more frequently in the wild but are not regarded to be the results of mutations. These include, for example, (1) the shape of the middle bar in *A. polymnus*, (2) the head bar in *A. nigripes* which can be joined dorsally or display a gap, and (3) the tendency of all three bars to disappear in a ventral to dorsal fashion in older individuals of *P. biaculeatus* and *A. ocellaris*. The last case may be linked to senescence related degeneration processes.

Research has suggested that approximately half of all coral reef fish species have evolved in the last 5 Myr [3]. While much of this massive radiation of new species is thought to have been driven by biogeographic conditions, colors and the patterns of fish are also thought to have played important roles [48], perhaps via stabilizing species boundaries [3]. Recent phylogenetic analyses of Amphiprioninae with molecular clock analyses show that a large majority of anemonefish species have also evolved within the last 5 Myr, likely due to biogeography combined with ecological speciation related to host-anemone associations [30, 32]. This Amphiprioninae phylogenetic framework combined with recent phylogenetic data on anemones [62] and the analyses of coloration and patterning as suggested in this review promise to open exciting new avenues of research that will allow a better understanding of the evolution of anemonefish pigmentation diversity.

## Concluding remarks on conservation issues

The conservation of coral reefs and therefore anemonefishes is an important topic facing critical problems [34]. It must be stated clearly that the introduction of non-native anemonefish species or mutants that have been artificially produced into coral reef ecosystems would be disastrous. Natural hybridization can play an important role in the evolution and radiation of taxa by increasing the genetic source [11, 16, 31], which is the case for the two naturally occurring hybrid species: *A. leucokranos* (*A. chrysopterus* x *A. sandaracinos*) and *A. thiellei* (*A. ocellari*s x *A. sandaracinos*). It seems unlikely that natural hybridization events will result in species with more complex color patterns, because all species of anemonefish show rather similar, “normal” color patterns. The highest degree imaginable is a hybrid with three horizontal bars and one dorsal stripe. However, the artificial breeding of hybrids is often seen as a negative when the conservation of wild populations is taken into regards [1]. Popular hybrid strains include “Blood Orange/Mai Tai” (*A. ocellaris* x *P. biaculeatus*), “Snow Onyx” (*A. ocellaris* “Snowflake” x *A. percula* “Onyx”), and “Black Photon” (*A. ocellaris* “Black Darwin” x *A. percula* “Onyx”) lines. Future breeding efforts, especially by aquaculture companies, will show if higher color pattern complexities of retail lines can be achieved; for example, (1) combining the “Lightning Maroon” phenotype of *P. biaculeatus* with *A. ocellaris*, or (2) introducing the “Snowflake” mutation of *A. ocellaris* into other anemonefish species. The introduction of hybrids into a natural system can lead to the loss of parental species, in particular when one of them is relatively rare. Moreover, if two species are bred that have similar morphological appearances, sometimes the offspring may not clearly be recognized as they are often an intermediate between the parent species. If such specimens are subsequently sold as a pure-bred species, breeding efforts to establish designer anemonefish, as well as conservation efforts, are threatened. Effective management of hybrids from a conservation perspective is still a topic under debate [1, 20, 50, 60]. Because the long-term outcomes of hybridization are unpredictable, care should be taken when acquiring and handling hybrids or suspected hybrids.

If these conservation issues are seriously taken, it must be emphasized that aquaculture trade anemonefish mutants offer a great entry point to better understand the genetics and development of complex and polymorphic pigmentation patterns. Anemonefish can be raised easily in laboratory husbandry systems and are open to developmental and genetic manipulation [38, 54] as well as to genomic analyses [54]. Therefore, identifying the genes affected in various mutants, manipulating them in laboratory environments, testing the effect of these mutations in the complex social system of captive anemonefish colonies, and studying the polymorphism and evolutionary patterns of these genes in wild populations will certainly shed light on how and why brilliant pigmentation patterns emerged in coral reef fishes.

## Supplementary Information

**Additional file 1:** Mutant anemonefish retail lines.

**Additional file 2:** Wild anemonefish variants.

## Data Availability

All data generated or analyzed during this study are included in this published article (and its Addditional files 1, 2).
